# Ecological study of *Paracoccidioides brasiliensis *in soil: growth ability, conidia production and molecular detection

**DOI:** 10.1186/1471-2180-7-92

**Published:** 2007-10-22

**Authors:** Gisela Ramos Terçarioli, Eduardo Bagagli, Gabriela Martins Reis, Raquel Cordeiro Theodoro, Sandra De Moraes Gimenes Bosco, Severino Assis da Graça Macoris, Virgínia Bodelão Richini-Pereira

**Affiliations:** 1 Departamento de Microbiologia e Imunologia, Instituto de Biociências de Botucatu, Universidade Estadual Paulista, Distrito de Rubião Júnior, s/n, 18618-000, Botucatu, São Paulo, Brasil

## Abstract

**Background:**

*Paracoccidioides brasiliensis *ecology is not completely understood, although several pieces of evidence point to the soil as its most probable habitat. The present study aimed to investigate the fungal growth, conidia production and molecular pathogen detection in different soil conditions.

**Methods:**

Soils samples of clayey, sandy and medium textures were collected from ground surface and the interior of armadillo burrows in a hyperendemic area of Paracoccidioidomycosis. *P*. *brasiliensis *was inoculated in soil with controlled humidity and in culture medium containing soil extracts. The molecular detection was carried out by Nested PCR, using panfungal and species specific primers from the ITS-5.8S rDNA region.

**Results:**

The soil texture does not affect fungus development and the growth is more abundant on/in soil saturated with water. Some soil samples inhibited the development of *P. brasiliensis*, especially those that contain high values of Exchangeable Aluminum (H+Al) in their composition. Some isolates produced a large number of conidia, mainly in soil-extract agar medium. The molecular detection was positive only in samples collected from armadillo burrows, both in sandy and clayey soil.

**Conclusion:**

*P. brasiliensis *may grow and produce the infectious conidia in sandy and clayey soil, containing high water content, mainly in wild animal burrows, but without high values of H+Al.

## Background

*Paracoccidioides brasiliensis *is the etiological agent of paracoccidioidomycosis (PCM), the most important systemic mycosis in Latin America [1]. While fungus can be isolated in its yeast form from patients, naturally infected armadillos and recently also dogs, the same does not hold true in nature where fungus may occur in its mycelial form producing the suspected infective propagules. The pathogen has been isolated only sporadically from soil and related materials, such as dog food and feces of bats and penguins [2-8]. The main route for infection seems to be by inhalation of airborne propagules, but the long latency period of the disease and the lack of epidemic outbreaks also create difficulties in determining under which circumstances the primary infection occurs [9]. The production of arthrospores *in vitro *has been documented [10], which also proved to be infectious in some animal models [11], but the number of these infectious structures has been observed to be very low and even absent for some isolates and/or culture conditions [10]. The influence of soil texture and chemical composition in fungus development has been little understood. Although in a same hyperendemic area infected armadillos occur both in sandy and clayey soil [12], the human infection seems to be more prevalent in regions where clayey soil is more abundant [13]. In this paper, we aimed to study the growth ability of fungus and its conidia production in different soil conditions, as well as fungus detection by PCR in soil collected from representative environmental conditions in an area hyperendemic for the disease.

## Methods

### Fungal isolates

Three *P. brasiliensis *isolates obtained from armadillos (T5LN1, T9B1 and T10B1) and three obtained from human patients (BT84, BT85 and D01) were maintained on Glucose Peptone Yeast Agar (GPYA: 2% Glucose, 1% Peptone, 2% Agar and 0.5% Yeast Extract) at 36°C in their yeast phase.

### Collection and analysis of soil macronutrients

**Figure 1:**
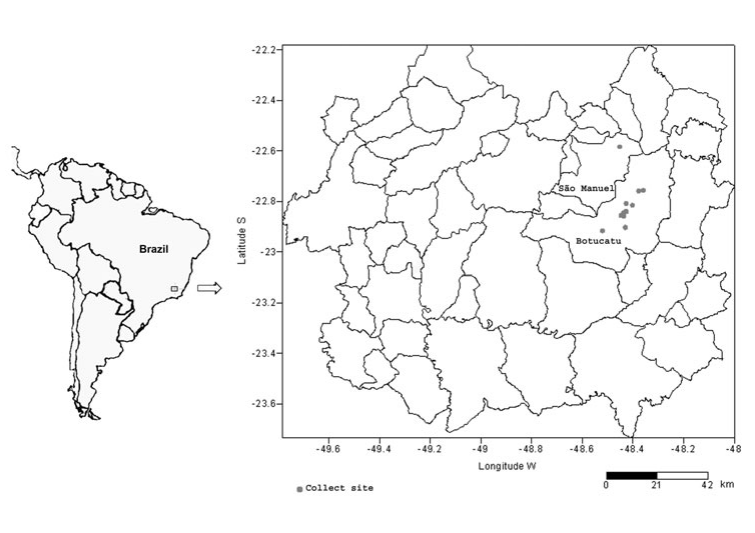
Geographic location of the collection sites in Botucatu hyperendemic area of paracoccidioidomycosis.

Soil samples of different textures (clayey, sandy and medium) were collected both from the surface and the interior of armadillo burrows located in Botucatu County and on a fluvial island in São Manuel County, both from the PCM hyperendemic area of Botucatu, Sao Paulo, Brazil. The collection sites were chosen according to the map of soil texture distribution for the region [14-16] and marked by GPS (Global Positioning System) (Figure 1). Representative samples from 9 different collection sites were obtained and sent for macronutrient analysis at the Soil Fertility Laboratory of the College of Agronomic Sciences-UNESP (Botucatu, São Paulo) (Table 1). The field capacity (FC), which indicates the amount of water present in soil after being saturated and drained of its excess, was also determined for each soil type [17]. Flasks containing around 500 g of each kind of soil texture were sterilized at 120°C for 30 min (twice), for further culturing of *P. brasiliensis*.

**Table 1:** Macronutrients analysis from soil samples collected in the PCM hyperendemic area of Botucatu.

**Collection sites**	**Geographic Coordinates**	**Texture**	**pH CaCl_2_**	**O.M g/dm^3^**	**P_resin _mg/dm^3^**	**CEC**	**K**	**Ca**	**Mg**	**BS**	**H+Al**	**V%**
							
						mmol_c_/dm^3^						
**Vitoriana road**	48°25'372"W 22°48'311"S	Clayey	5.3	63	43	153	4.5	74	31	110	43	72
**Capivarinha river/Vitoriana District**	48°21'993"W 22°45'327"S	Sandy	4.6	29	31	54	0.9	18	9	28	26	52
**Vitoriana pasture**	48°21'363"W 22°45'247"S	Sandy	4.0	19	27	30	0.8	2	3	5	24	18
**Lageado farm**	48°25'389"W 22°50'219"S	Clayey	6.0	54	38	138	0.7	86	29	115	23	83
**Lavapés river**	48°25'730"W 22°50'505"S	Clayey	4.7	49	56	150	3.5	51	27	82	68	55
**Patrulha, Lageado**	48°26'051"W 22°51'226"S	Medium	3.9	42	28	101	0.5	1	1	2	98	2
**Botanic garden**	48°30'075"W 22°53'11.9"S	Sandy	3.9	19	28	45	0.4	3	3	6	39	14
**Serrito Island A**	48°25'371"W 22°34'659"S	Medium	6.6	25	29	105	0.6	2	2	5	100	5
**Serrito Island B**	48°25'484"W 22°34'366"S	Medium	3.7	23	39	77	0.5	2	2	4	73	5

O.M.: Organic Matter, CEC: Cation Exchange Capacity, BS: Sum of Exchangeable Bases (Ca + Mg + K), H+Al: Exchangeable Aluminum, V%: Base Saturation.

### Culturing of *P. brasiliensis *on soil

Approximately fifty grams of sterilized soil (clayey and sandy) saturated with distilled water was placed into Petri Dishes, where four yeast fragments were inoculated and maintained at 25°C.

### Culturing of *P. brasiliensis *on soil with controlled humidity

The soil samples were dried at 120°C for 24 h. Fifty grams of this soil was placed into Petri Dishes and sterilized at 120°C for 15 min. Depending on the FC of each soil, different volumes of sterile distilled water were added to reach three different humidity degrees: low (soil with half of its FC), medium (soil with one whole FC) and high (soil saturated in water). For this experiment, two yeast fragments of isolates T5LN1, T9B1 and BT84 were cultured for each soil texture and maintained at 25°C.

### Culturing of *P. brasiliensis *in Soil Extract Agar (SEA), Potato Dextrose Agar (PDA) and GPY Agar (GPYA) plus Soil Extract (SE)

Soil extract from different soil textures and compositions were prepared by mixing 1 kg of soil with 1L of distilled water, sterilized in an autoclave 120°C for 30 min, filtered in a glass funnel containing gauze plus cotton and pH adjusted to 7.0. The soil extracts were prepared with samples of sandy SE collected from the Capivarinha River, of clayey SE from Lageado Farm and of medium SE from Patrulha (Table 1). The SEA (0.2% Glucose, 0.1% Yeast extract, 1.5% Agar and 50% v/v of SE) was prepared according to Kwon-Chung [18]. For the PDA (OXOID) and GPYA (2% Glucose, 0.5% Peptone, 0.5% Yeast extract, 1.5% Agar), the SE was added at 30% v/v. Four yeast fragments were seeded in duplicate plates that were maintained at 25°C. For this experiment five isolates were used: BT84, D01, T5LN1, T9B1 and T10B1.

### Growth ability of *P. brasiliensis*

For each treatment, the fungal growth was evaluated macroscopically for 8 weeks by measuring the diameter of colonies in centimeters.

### Conidia production

The conidia production of the isolates BT84, BT85, D01, T5LN1, T9B1 and T10B1 was evaluated microscopically by the slide culture technique and also by using adhesive tape (Durex, 3 M), which was softly touched onto the surface of the colonies, previously killed by formaldehyde vapor for 48 h. The slides were stained with Lactophenol cotton blue. For some productive conidia isolates, we also compared the number of conidia between SEA and GPYA with SE, by counting at least 10 different microscopic slide fields. The slides were observed and documented in an Olympus Photomicrographic System (PM-30), as well as by Leica Digital Photomicrography (DMLB) with the software Leica Qwin Lite 2.5 to estimate the conidium sizes.

### Statistical analysis

The number of conidia per field in different conditions and the measure of mycelial growth of *P. brasiliensis *in SEA, GPYA and PDA plus SE were analyzed through ANOVA followed by Tukey-Kramer (software GraphPad InStat).

### DNA extraction

The DNA was extracted from the yeast phase of *P. brasiliensis *(positive control) according to Van Burik et al. [19]. The DNA extraction from soil seeded with *P. brasiliensis *(positive control) and from environmental samples was carried out by using Fast DNA^® ^Spin Kit for Soil (Qbio gene INC), with minor modifications according to Theodoro et al.[20]. A total of 21 crude environmental soil samples of different textures (clayey, sandy and medium) collected both from the surface (4 samples) and the interior of armadillo burrows (17 samples) were processed.

### Nested PCR from soil samples

The molecular detection was carried out by Nested-PCR reactions, using the panfungal primers ITS4 and ITS5 as outer primers [21] and PbITSE and PbITSR as inner primers [20]. The reactions were carried out in 25 μl of reaction mixture (10 ng of genomic DNA, 1× PCR buffer – 50 mM KCL and 10 mM Tris HCL -, 1.5 mM MgCl_2_, 0.2 mM dNTP, 10 pmoles of each primer and 1 unit of *Taq *polymerase-Amersham Biosciences/GE Healthcare). The amplification was done in a thermocycler (MJ Research, Inc, USA). The thermal cycling for ITS4/ITS5 was: 94°C for 5 min followed by 25 cycles of 94°C for 1 min, 60°C for 2 min, 72°C for 2 min and a final cycle at 72°C for 7 min. For PbITSE/PbITSR primers the conditions were similar, except for the annealing temperature which was 62°C [20].

Two amplicons from the Capivarinha River and two from Lageado Farm, all from burrows, were purified with GFX PCR DNA and gel band purification kit (Amershan Biosciences) and both strands were directly sequenced in a Mega Bace™ 1000 (Amershan Biosciences) with reactions performed according to DYEnamic ET Dye Terminator Cycle Sequencing Kit (Amershan Biosciences). The sequences were submitted to Blastn (Basic Local Alignment Tool for Nucleotide) and aligned using Clustal W program.

## Results

### *P. brasiliensis *growth ability in soil with different chemical and physical compositions

Macronutrient analyses of soil samples are in Table 1. The fungal growth was similar in clayey and sandy soil textures. No growth was observed when the soil composition contained high values of Exchangeable Aluminum (H+Al) and, consequently, a low Bases Saturation value (V%), such as those from Patrulha Lageado and Serrito Island (Table 2). Most of the isolates presented the yeast-mycelia transition in the first week and had reached high mycelial growth by the 8^th ^week. The isolate BT84 did not grow in any soil sample. There were some differences among the isolates concerning the growth and macroscopic mycelial morphology (Table 2).

**Table 2:** Growth ability of *Paracoccidioides brasiliensis *in soil and Soil Extract Agar (SEA) from different textures.

		**Sandy Soil**		**Clayey Soil**		Medium Soil*	
**Isolates**	Weeks	SEA	SOIL	SEA	SOIL	SEA	SOIL
**T5LN1**	1	-	+	-	+	-	-
	2	++	++	++	++	++	-
	4	+++	+++	+++	++	++	-
	8	++++	++++	+++	++	++	-
**T9B1**	1	-	+	-	+	-	-
	2	++	++	++	++	++	-
	4	++++	+++	+++	+++	+++	-
	8	++++	++++	++++	++++	++++	-
**T10B1**	1	-	-	-	-	-	-
	2	-	-	+	+	+	-
	4	+++	++	+++	+++	+++	-
	8	++++	++++	++++	++++	++++	-
**D01**	1	-	+	-	+	-	-
	2	+	++	++	+++	+	-
	4	+++	+++	++	+++	++	-
	8	+++	++++	+++	++++	+++	-
**BT84**	1	-	-	-	-	-	-
	2	+	-	++	-	+	-
	4	+++	-	++	-	++	-
	8	+++	-	++	-	++	-

"+" transformation from yeast to mycelial form; "++" low mycelial growth (up to 1.25 cm diameter); "+++" medium mycelial growth (from 1.25 to 1.65 cm diameter); "++++" high mycelial growth (above 1.65 cm diameter); "-" no growth.

*The samples from medium textures presented high values of H+Al and low values of base saturation.

### Effect of soil humidity on *P. brasiliensis *growth

The fungal growth was higher in soil saturated with water than in soil with moderate humidity, determined by its field capacity (FC). There was no growth on low humidity soil, such as in the one that contains only half of its FC (Table 3).

**Table 3:** Growth analysis of *Paracoccidioides brasiliensis *in soil with humidity control and maintained at 25°C.

		**Weeks of Growth**							
		
		Sandy Soil				Clayey Soil			
**Humidity**	**Isolate**	1	2	4	8	1	2	4	8
**Low (soil in half FC)**	T5LN1	-	-	-	-	-	-	-	-
	T9B1	-	-	-	-	-	-	-	-
**Medium (soil in FC)**	T5LN1	-	+	+	+	-	-	+	+
	T9B1	-	+	+	++	-	+	+	++
**High (saturated soil)**	T5LN1	+	++	++	++	-	+	++	++
	T9B1	+	++	++	++	+	++	++	++++

"+" transformation from yeast to mycelial form; "++" low mycelial growth (up to 1.25 cm diameter); "+++" medium mycelial growth (from 1.25 to 1.65 cm diameter); "++++" high mycelial growth (above 1.65 cm diameter); "-" no growth.

### Culturing of *P. brasiliensis *in SEA and in GPYA and PDA plus SE

**Figure 2:**
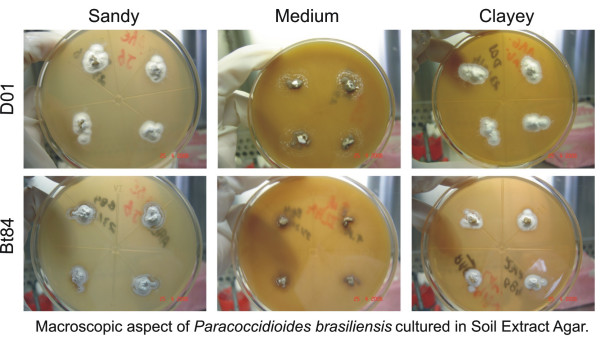
Macroscopic aspect of *Paracoccidioides brasiliensis *cultured in Soil Extract Agar (SEA) prepared with different soil textures: sandy (first column), medium that contains high amount of H+Al (second column) and clayey (third column). Isolates D01 (A-C) and Bt84 (D-F).

Most fragments cultured on SEA presented the yeast-mycelia transition during the second week. The isolate BT84 presented the smallest growth in the two soil textures. The isolates T9B1 and T10B1 attained high mycelial growth in the 8^th ^week while the isolates T5LN1 and D01 grew only moderately during this period (Table 2). SE prepared from clayey or sandy soil tended to induce the same growth, but when prepared with soil containing high values of H+Al, the growth also occurred, although at low intensity for most isolates and the colonies showing a glabrous aspect (Figure 2).

The addition of SE on GPYA and PDA seemed to have had no effect on fungal growth.

### Conidia production

**Figure 3:**
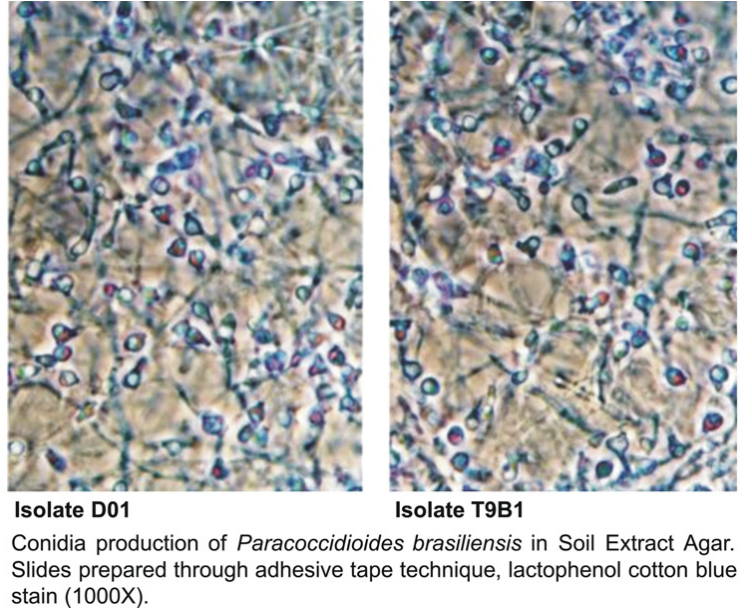
Conidia production of *Paracoccidioides brasiliensis *in Soil Extract Agar, exhibiting the conidia production of the isolates D01 and T9B1 cultured in soil extract agar (SEA) and prepared through adhesive tape technique (magnification ×1000).

The isolates T9B1, BT85 and D01 produced a large amount of arthro- and aleuroconidia in the different SEA (Figure 3), while the isolates T5LN1, T10B1 and BT84 did not produce conidia. The adhesive tape slides stained with lactophenol cotton blue showed more conidia per field than the slide culture technique (p < 0.05).

The isolates T9B1 and D01 produced fewer conidia in GPYA plus SE than in SEA (p < 0.05) and the isolate Bt85 did not produce any conidia in GPYA plus SE in slide culture.

The width and length of conidia were 2.88 μm ± 0.57 and 3.49 μm ± 0.69, respectively.

### Molecular detection of *P. brasiliensis *in soil samples

**Figure 4:**
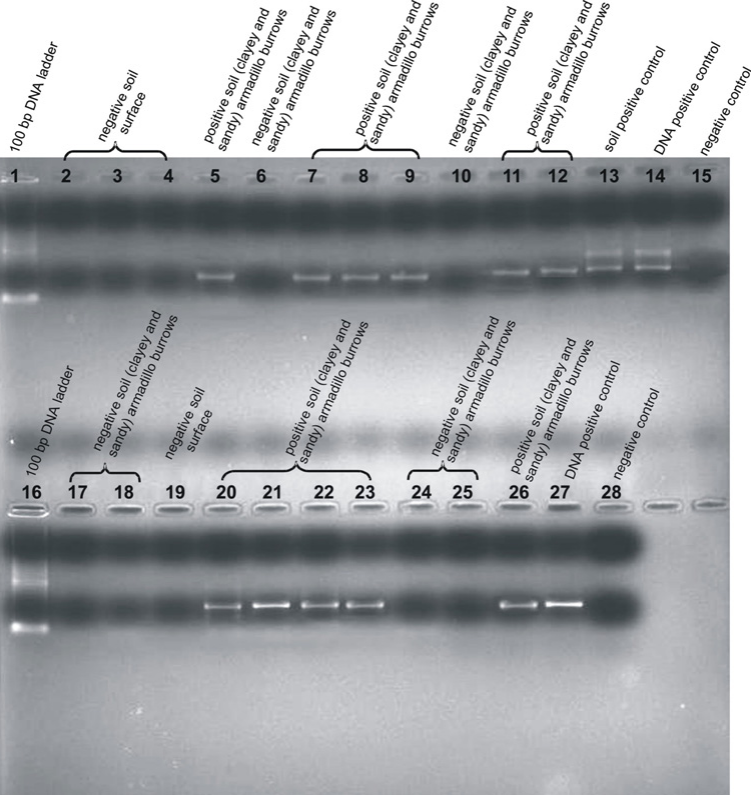
Nested PCR from soil samples with inner primers PbITSE and PbITSR. Lanes 1 and 16: 100 bp DNA ladder; Lane 13: soil seeded with *P. brasiliensis *(soil positive control); Lanes 14 and 27: DNA positive control; Lanes 15 and 28: negative control; Lanes 5, 7, 8, 9, 11, 12, 20, 21, 22, 23 and 26: positive soil samples (clayey and sandy) collected from armadillo burrows. Lanes 2, 3, 4 and 19: negative soil samples collected from surface; Lanes 6, 10, 17, 18, 24 and 25: negative soil samples collected from armadillo burrows.

The Nested PCR reactions were positive for most soil samples from armadillo burrows (both clayey and sandy textures). There was no positive amplification for the surface soil samples (Figure 4). Direct sequencing of ITS amplicons (from clayey and sandy soil) showed 100% similarity with the deposited sequences (gi|52078067|gb|AY631237.1|) of *P. brasiliensis *at GenBank.

## Discussion

Although some ecological aspects of *P. brasiliensis *are still unknown, several pieces of evidence point to the soil as its main saprobic habitat. Other related thermo-dimorphic species, such as *Blastomyces dermatitidis, Coccidioides immitis *and *Histoplasma capsulatum*, have already been determined to be associated with soil [22-24]. In our paracoccidioidomycosis hyperendemic area, a high number of human cases seem to be associated with clayey soil areas, while infected armadillos have been observed also in sandy soil [12,13]. We observed here that fungus can develop both on clayey and sandy soils when in high humidity. Thus, the major incidence of paracoccidioidomycosis in clayey soil areas may be due to the widespread use of this soil type in agricultural activity by rural workers that represent most disease cases. The data also confirm that humidity is an important ecological factor for fungus occurrence in soil. It was already suggested that the presence of the pathogen in the environment may be associated with high amounts of rainfall and abundant watercourses [25]. Some soil conditions, in particular that contains elevated Exchangeable Aluminum (H+Al) may inhibit or limit fungus growth. This fact was observed in some soil samples that, in this study, were the same as medium-textured soils. High values of Exchangeable Aluminum determine low values of Base Saturation, since they are connected and inversely proportional [26]. These data also corroborate the results of *P. brasiliensis *isolation from armadillos in the same endemic area. While at most capture sites the animals were culture positive, in one restricted area with high levels of Exchangeable Aluminum, all evaluated armadillos were culture negative [12].

It is important to emphasize that the paracoccidioidomycosis "reservarea", i.e., the location where the pathogen habitat and infection coincide, is influenced by several abiotic factors. For example, water availability depends on soil characteristics, precipitation and evaporation rate. Under the same climatic conditions, clayey soils can retain more water and nutrients than sandy ones, due to the greater surface area per mass unit of clay particles [13]. Although we observed that the soil texture was not directly responsible for the fungal growth in the environment, the local climatic conditions associated with the soil texture would determine the development of the pathogen in its saprobic phase.

Some isolates could be more able to develop in their saprobic phase, such as T9B1 which presented the highest growth in all conditions, while other isolates could disperse more slowly and produce few or no conidia, such as BT84 which presented the least development and did not produce any propagula. Conidia production in *P. brasiliensis *is considered to be rare and difficult to obtain in the traditional culture conditions. Restrepo and colleagues [27,28] induced its production by adding natural substrates in the media, such as Soil Extract Agar SEA. Conidia production was also induced in poor media such as yeast extract, glucose-salts and water-agar media. The SEA media proved to be very efficient for the conidia study due to their large recovery, which was greater than in GPY Agar plus soil extract. The morphological aspects and measures of the conidia observed herein were similar to some previous studies and compatible with airborne infection [10].

Experimental models of infection by conidia, by the intranasal route, in mice, showed that these propagules have infectious potential, being able to initiate paracoccidioidomycosis disease [11]. Furthermore, the conidium consists of a eukaryotic cell, containing nutrient reserves, which would allow its survival in the environment [29]. Due to its thermodimorphic property, a conidium can develop into both a mycelial and yeast phase, depending on the substrate (saprobic or host tissues) [30]. Since conidia production seems to be an important feature for *P. brasiliensis *infection and dissemination in animal hosts, the SEA media can be used as an important tool for the study of the interaction of this pathogen with its microhabitat.

There were some differences among the isolates both in growth ability and conidia production. For example, one isolate (BT84) practically does not grow in soil while all the others growth well. Some isolates did not produce conidia while in others the conidia production was relatively high, mainly when the fungus was grown on relatively poor substrates containing soil extracts. These features could be associated with the genetic background of the isolates and also related to the evolutionary history of the pathogen. For example, the isolates T10B1 and BT84, which are considered to be from the same phylogenetic species (PS2) group, according to Matute et al. (2005) [31], did not produce any conidia, while in the isolates T9B1, BT85 and T5LN1, which belong to the S1 species group, significant conidia production is present. This feature (conidia production) may be taken into account as a morpho/physiological marker in order to help distinguish the several *P. brasiliensis *cryptic species.

The positive Nested PCR in soil samples from armadillo burrows and the negative amplifications from surface soil samples reinforce the idea that the pathogen habitat is strongly related to the presence of the 9-banded armadillo [12]. It is possible that the soil from inside the burrows is not submitted to great variations in humidity, temperature, shade and other abiotic factors, compared to the surface soil. Thus the armadillo burrows and their surrounding areas must be considered important sites of infection risk, which would explain the high paracoccidioidomycosis incidence in patients who had some contact with this animal [25].

The characterization of the saprobic phase of *P. brasiliensis*, concerning its soil growth ability and conidia production, developed in this study can be useful for a better understanding of the pathogen ecology as well as for further studies on morpho/physiological differences among the several new cryptic species. Besides that, our results proved the repeatability and applicability of the molecular technique, previously developed in our laboratory, to several environmental samples, identifying some infection risk areas for human beings.

## Conclusion

While soil texture is not a determinative factor for the development of *Paracoccidioides brasiliensis*, humidity plays an important role in fungus growth. Soils containing high amounts of H+Al might inhibit or limit the growth of the pathogen. The conidia production of *P. brasiliensis *is dependent on the isolate genotype and is produced in especially high amounts in substrates containing soil extracts. Specific molecular detection of the pathogen indicates *P. brasiliensis *may occur preferentially in soils from protected places such as from the interior of armadillo burrows. These findings may contribute to identifying places where the pathogen occurs in the environment, helping us to map risk areas for infection while contributing to the understanding of the fungus ecology and disease epidemiology.

## Competing interests

The author(s) declare that they have no competing interests.

## Authors' contributions

GRT: Study design, fieldwork and data collection, laboratory tests, statistics, data interpretation and analysis, manuscript writing.

EB: Coordination, study design, fieldwork and data collection, data interpretation and analysis, manuscript writing.

GMT: Laboratory tests.

RCT: Study design, fieldwork and data collection, data interpretation and analysis, manuscript writing.

SMGB: Data interpretation and analysis, manuscript writing.

SAGM: Laboratory tests.

VBR: Fieldwork and data collection, statistics, data interpretation and analysis, manuscript writing.

All authors have read and approved the final version of the manuscript.
